# Analysis of Morphological High-Risk Factors for Retrograde Type A Aortic Dissection

**DOI:** 10.31083/RCM42881

**Published:** 2026-04-08

**Authors:** Chaozhong Long, Jinhai Xia, Chaoen Luo, Jinzhou Cai, Asfandyar Khan, Yaoguang Feng, Zhengwen Lei

**Affiliations:** ^1^Department of Cardiothoracic Surgery, The First Affiliated Hospital, Hengyang Medical School, University of South China, 421009 Hengyang, Hunan, China; ^2^Department of Thoracic Surgery, Yueyang Central Hospital, 414000 Yueyang, Hunan, China; ^3^Department of Thoracic Surgery, Yueyang People’s Hospital, Hunan Normal University, 414000 Yueyang, Hunan, China

**Keywords:** RTAD, TBAD, retrograde tear, morphological high-risk factors, risk prediction model

## Abstract

**Background:**

The mechanism involved in retrograde type A aortic dissection (RTAD) remains unclear, while research through morphological studies is limited. Therefore, this study aimed to compare the aortic geometric features between RTAD and type B aortic dissection (TBAD) and to identify specific anatomical predictors of RTAD.

**Methods:**

A total of 60 patients diagnosed with acute aortic dissection, with the primary entry tear located in the descending aorta, were included based on aortic computed tomography angiography (CTA) performed at our center between November 2019 and November 2023. Among them, 21 were RTAD cases, and 39 were TBAD cases. Aortic CTA morphological data were collected using Carestream Image Suite V4 and EndoSize software. Retrospective statistical analysis was performed using SPSS 26 and RStudio to explore relationships among aortic and aortic arch morphologies, angles, primary tear location, tear size, and dissection type.

**Results:**

(1) No significant differences were observed between the two groups in gender, age, height, weight, body mass index (BMI), hyperlipidemia, hypertension, diabetes, coronary artery disease, or smoking history (all *p* > 0.05). (2) Multivariate logistic analysis revealed that reduced minimum diameter of the ascending aorta (odds ratio (OR) 0.488, 95% confidence interval (CI) 0.245–0.974; *p* = 0.042), increased maximum diameter of the ascending aorta (OR 2.318, 95% CI 1.107–4.857; *p* = 0.026), and reduced minimum diameter of the distal aortic arch (OR 0.594, 95% CI 0.362–0.974; *p* = 0.039) were significant predictors of RTAD. (3) The RTAD risk prediction model demonstrated excellent predictive performance and robustness across datasets and experimental conditions, effectively identifying high-risk patients and providing reliable support for clinical decision-making (C-index = 0.952; area under the curve (AUC) = 0.952). Calibration curves showed high consistency between predicted probabilities and observed outcomes, and decision curve analysis (DCA) indicated significant clinical net benefits across a wide range of threshold probabilities.

**Conclusions:**

(1) Reduced minimum diameter of the ascending aorta, increased maximum diameter of the ascending aorta, and reduced minimum diameter of the distal aortic arch are specific predictors of TBAD progressing to RTAD. (2) The RTAD risk prediction model, incorporating these high-risk factors, offers clinical guidance for the prevention and early intervention of RTAD.

## 1. Introduction

**Fig. 1.**
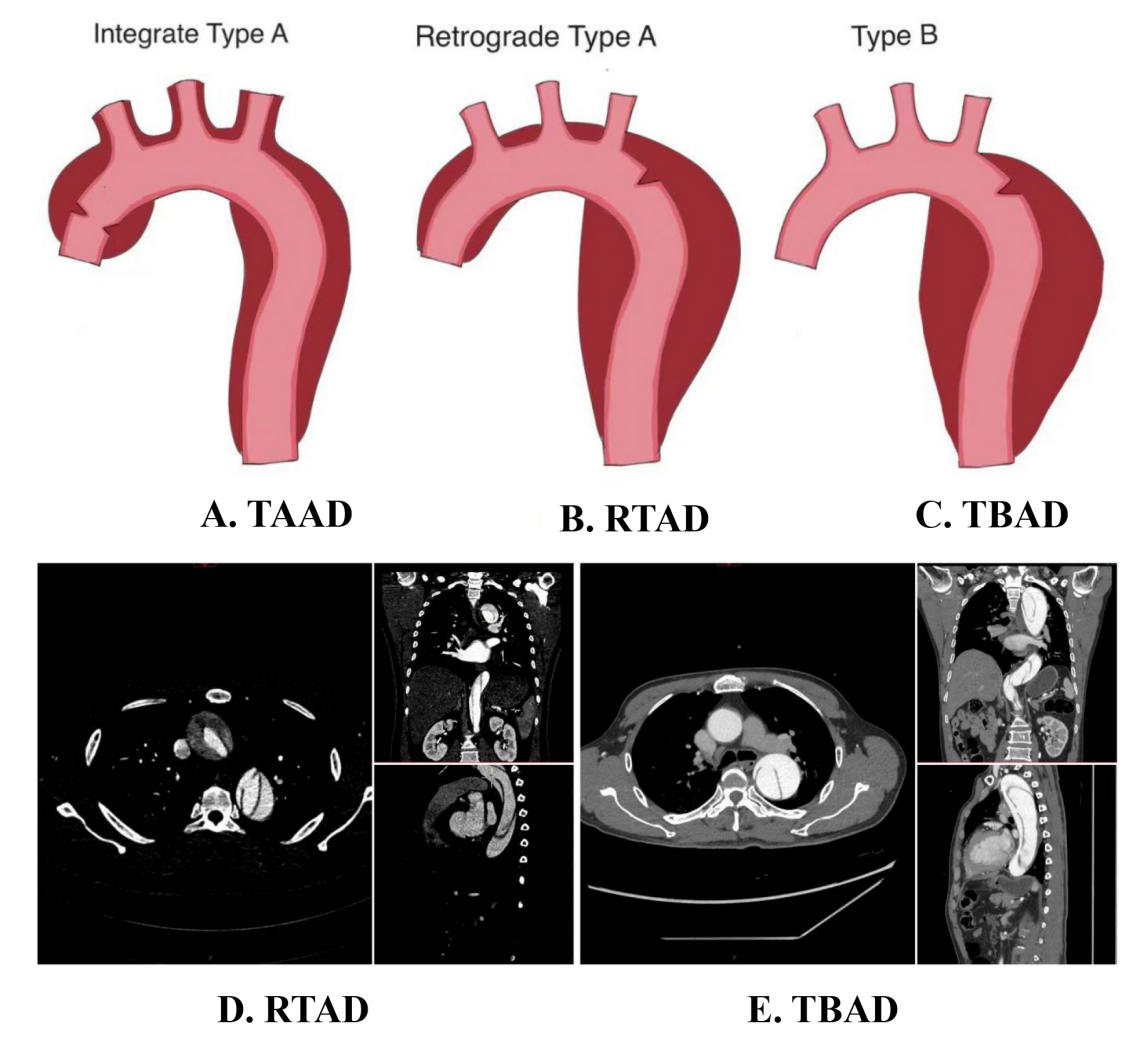
**Schematic diagrams of different types of aortic dissection**. (A) Tear location and extent of involvement in TAAD. (B) Tear location and extent of involvement in RTAD. (C) Tear location and extent of involvement in TBAD. (D) CTA imaging of RTAD in axial, coronal, and sagittal planes. (E) CTA imaging of TBAD in axial, coronal, and sagittal planes. TAAD, type A aortic dissection; RTAD, retrograde type A aortic dissection; TBAD, type B aortic dissection; CTA, computed tomography angiography.

Aortic dissection is an acute, rapidly progressing, and life-threatening cardiovascular emergency characterized by a tear in the aortic intima, allowing blood to enter the medial layer and form true and false lumens. Based on the tear location and extent, aortic dissection is classified into two main types. The DeBakey classification divides it into types I (originating in the ascending aorta and extending to the arch and descending aorta), II (confined to the ascending aorta), and III (originating in the descending aorta). The Stanford classification categorizes it as type A (involving the ascending aorta) or type B (not involving the ascending aorta), with Stanford A encompassing DeBakey I/II and Stanford B corresponding to DeBakey III. Type A dissections are more common and clinically critical [1, 2]. The estimated annual incidence of aortic dissection is 2.5–3.5 per 100,000 individuals. Approximately 65% originate in the ascending aorta, 20% in the descending aorta, 10% in the aortic arch, and 5% in the abdominal aorta. It predominantly affects males (65%) aged 60–70 years [3, 4, 5, 6]. Due to its unique retrograde propagation characteristics, retrograde type A aortic dissection (RTAD) is relatively uncommon, accounting for 5% to 25% of Type A aortic dissections [7, 8]. RTAD represents a special subset of type A aortic dissection (TAAD), where the primary intimal tear originates distal to the left subclavian artery and propagates retrograde to involve the ascending aorta. RTAD constitutes approximately 9% of TAAD cases [9]. Sun Lizhong [10] proposed that RTAD represents a rare subtype of aortic dissection (Fig. 1), typically originating in the descending aorta and propagating retrogradely to the ascending aorta, classified as the “AC type” (complex Type A) in refined aortic dissection classifications due to its involvement of the aortic arch and branch arteries. Owing to its retrograde trajectory and invasion of the ascending aorta, RTAD poses significant therapeutic challenges, with high mortality rates and ongoing debates regarding optimal management [11]. Some studies suggest that RTAD patients may have better prognoses than classic TAAD patients, advocating for endovascular therapy or medical management [12, 13, 14]. Conversely, other experts recommend aggressive surgical intervention, including total arch replacement with frozen elephant trunk (FET) repair, combined with distal malperfusion correction and primary entry tear exclusion to prevent early- and mid-term complications [15, 16]. Morphological differences between dissection types influence pathophysiology, diagnosis, and treatment. RTAD-specific features may include tear location, false lumen characteristics, and hemodynamic variations [17, 18, 19, 20, 21]. However, research on RTAD morphology remains limited compared to clinical and therapeutic studies. This study compares RTAD and type B aortic dissection (TBAD) patients to identify RTAD-specific morphological risk factors, aiming to enhance clinical decision-making and prognosis.

## 2. Materials and Methods

### 2.1 Study Population

This retrospective study analyzed 60 patients diagnosed with aortic dissection between November 2019 and November 2023, all with primary entry tears in the descending aorta. In the study design, we used a sample size estimation formula for binary variables based on the incidence rates reported in previous studies. We calculated with an alpha of 0.05 and a power of 0.80, and the results showed that at least 20 patients per group would meet the requirements for model analysis. The study ultimately included 21 patients with RTAD and 39 with TBAD. Demographic and clinical data, including gender, age, height, weight, body mass index (BMI), clinical presentation, medical history (e.g., hyperlipidemia, hypertension, diabetes, coronary artery disease), and prior cardiac/thoracic trauma/surgery, were collected from medical records. Aortic morphological parameters were measured using Carestream Image Suite V4 (New York, USA) and EndoSize (Endovastec, Shanghai, China) software, including:

(1) Maximum/minimum diameters of the aortic root, ascending aorta, proximal/distal aortic arch, and true/false lumen areas. (2) Aortic arch morphology, entry tear location (greater/lesser curvature, anterior/posterior wall), tear size (length × width), and distance from the tear to the left subclavian artery. (3) Maximum/minimum diameters and true/false lumen areas at the proximal tear site, tear site, distal tear site, celiac trunk level, renal artery level, and aortic bifurcation. (4) Aortic arch angles (α and β angles). The study was approved by the Hospital Ethics Committee (Ethics No.: 2024LL0129002), with informed consent obtained from all patients and families.

### 2.2 Inclusion and Exclusion Criteria

#### 2.2.1 Inclusion Criteria

(1) Confirmed diagnosis of aortic dissection via preoperative imaging (computed tomography angiography (CTA), MRI, or ultrasound). (2) Complete imaging data (dissection length, thickness, tear location, true/false lumen ratio). (3) RTAD group: Dissection involving the ascending aorta but originating in the descending aorta, with no tears in the ascending aorta/aortic arch and primary tear located distal to the left subclavian artery. (4) Acute phase (symptom onset <14 days).

#### 2.2.2 Exclusion Criteria

(1) Chronic phase (symptom onset >14 days). (2) Severe comorbidities affecting outcomes (e.g., acute myocardial infarction, severe infection, end-stage cancer, renal failure). (3) Incomplete imaging data or lack of informed consent. (4) History of cardiac/thoracic surgery, trauma, immune/connective tissue disorders, or valvular abnormalities on echocardiography. (5) Secondary RTAD (e.g., post-thoracic endovascular aortic repair).

#### 2.2.3 Flow Chart for Patient Selection

**Fig. 2.**
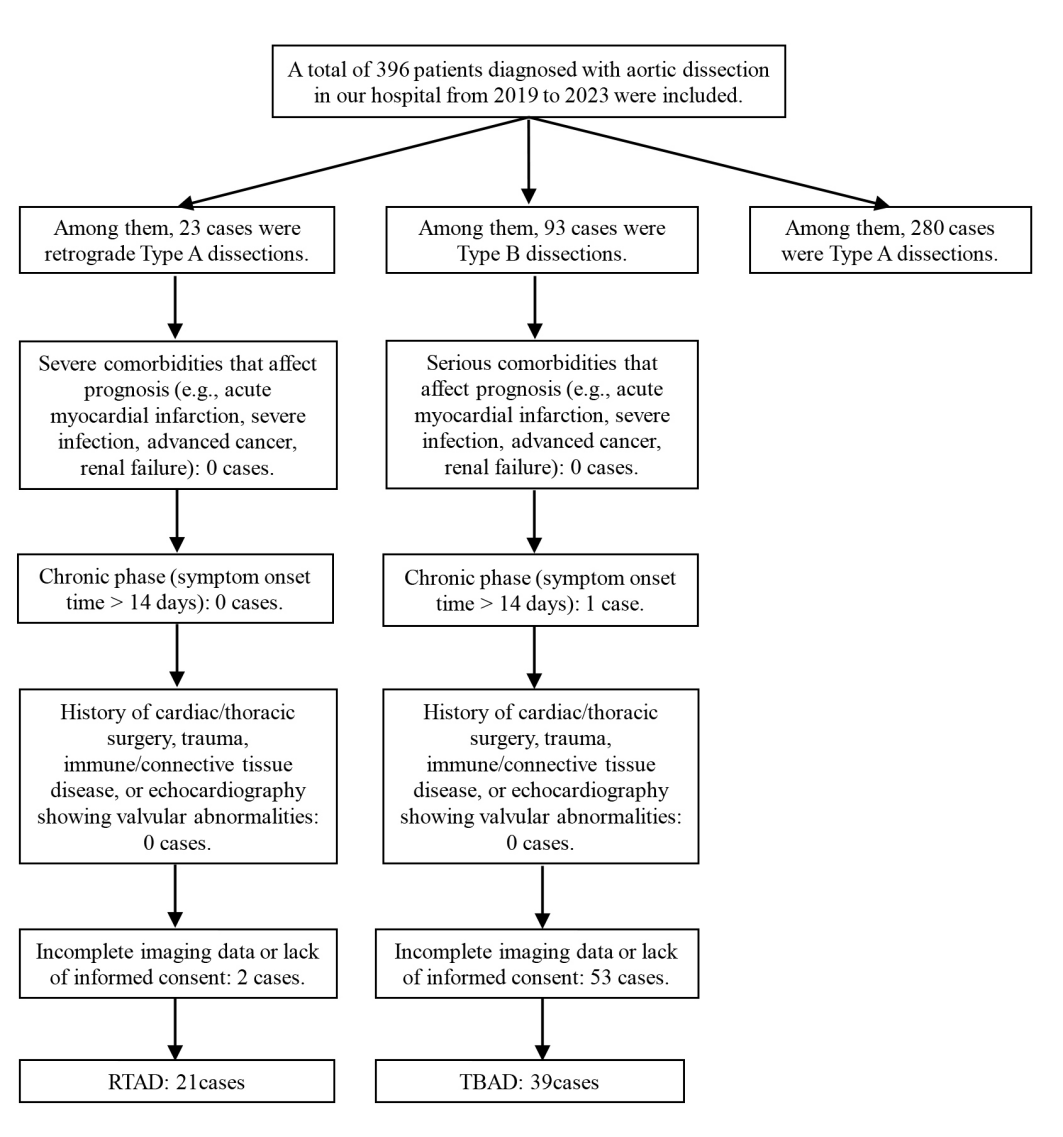
**Flow Chart for Patient Selection**.

The study selection process is shown in Fig. 2.

### 2.3 Clinical Data Collection

#### 2.3.1 Baseline Data

Gender, age, height, weight, BMI, hyperlipidemia, hypertension, diabetes, coronary artery disease, and smoking history.

#### 2.3.2 Study Protocol

**Fig. 3.**
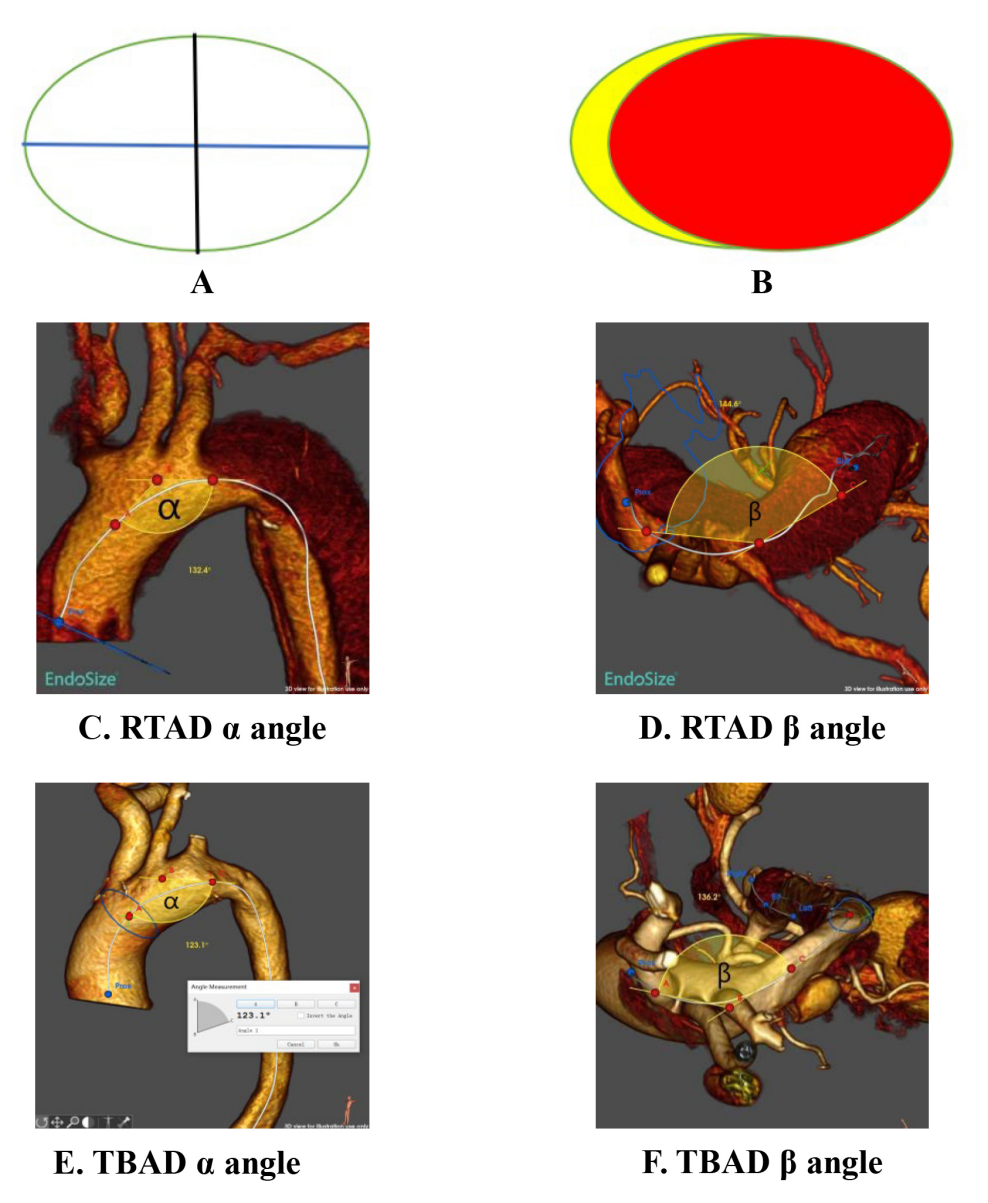
**Schematic diagram of measurement methods for aortic diameter, angle, and area**. (A) Black line: Minimum diameter; Blue line: Maximum diameter. (B) Yellow: False lumen area; Red: True lumen area. (C) Maximum projection direction of the aortic arch (alpha angle, α) in the left anterior oblique (LAO) view of RTAD. (D) Aortic centerline angle and beta angle (β) in the superior view of RTAD. (E) Maximum projection direction of the aortic arch (alpha angle, α) in LAO view of TBAD. (F) Aortic centerline angle and beta angle (β) in the superior view of RTAD.

Statistical Analysis: IBM SPSS Statistics 26.0 (Armonk, New York, USA) was used to compare morphological parameters between groups, including aortic diameters, true/false lumen areas, tear characteristics, and aortic arch angles (Fig. 3). Least Absolute Shrinkage and Selection Operator (LASSO) Regression: Dimensionality reduction was applied to identify significant variables. Multivariate Logistic Analysis: Predictive factors were incorporated into a risk model to identify RTAD-specific high-risk factors. Imaging Analysis: Measurements were independently performed by two senior radiologists and one cardiothoracic surgeon to ensure consistency.

#### 2.3.3 Angle Measurement Methods

(1) α Angle (Left Anterior Oblique Projection): Defined as the angle between the tangent lines of the proximal brachiocephalic trunk and distal left subclavian artery along the aortic centerline in the left anterior oblique (LAO) projection. (2) β Angle (Superior View): Measured as the angle of the aortic centerline in the superior view.

Procedure: The aortic centerline was reconstructed using EndoSize software. The α angle was measured in the LAO projection (180°), and the β angle was measured in the superior view (90° cranial angulation).

### 2.4 Statistical Analysis

Continuous variables: Normally distributed data were expressed as mean ± standard deviation and compared using independent *t*-tests. Non-normally distributed data were presented as median (interquartile range) and analyzed with nonparametric tests. Categorical variables: Described as frequency (%) and compared using chi-square tests. Variable selection: LASSO regression was applied for dimensionality reduction. Variables with statistical significance in LASSO analysis were incorporated into a multivariate logistic regression model. Odds ratios (ORs) with 95% confidence intervals (CIs) were calculated, and a nomogram was constructed to visualize the prediction model. Model validation: The concordance index (C-index), receiver operating characteristic (ROC) curve, calibration curve, and decision curve analysis (DCA) were performed for internal validation. A two-tailed *p* < 0.05 was considered statistically significant.

### 2.5 In This Study, the Measurement of Aortic Diameter Included Several Key Locations

#### 2.5.1 Aortic Root

The diameter was measured at the aortic root, specifically at the sinus of Valsalva.

#### 2.5.2 Ascending Aorta 

Both the maximum and minimum diameters were measured in the upper segment of the ascending aorta.

#### 2.5.3 Aortic Arch

Based on the anatomical structure of the aortic arch, we defined two measurement levels for the proximal and distal segments. The proximal measurement was taken near the origin of the brachiocephalic artery, and the distal measurement was taken near the origin of the left subclavian artery.

## 3. Results

### 3.1 Baseline Characteristics

The study included 60 patients: 39 in the TBAD group (32 males, 7 females; mean age 54.18 ± 13.78 years) and 21 in the RTAD group (20 males, 1 female; mean age 49.33 ± 10.03 years). No significant differences were observed between the two groups in gender, age, height, weight, BMI, hyperlipidemia, hypertension, diabetes, coronary artery disease, or smoking history (all *p* > 0.05). Details are shown in Table 1.

**Table 1.** **Comparison of general characteristics between groups**.

Clinical data		TBAD (n = 39)	RTAD (n = 21)	t/χ^2^ value	*p*-value
Sex				1.071	0.301
	Male	32 (82.1%)	20 (95.2%)		
	Female	7 (17.9%)	1 (4.8%)		
Age		54.18 ± 13.78	49.33 ± 10.03	1.420	0.161
Height		168.72 ± 5.09	168.57 ± 6.42	0.097	0.923
Weight (kg)		71.26 ± 9.64	71.14 ± 12.11	0.040	0.968
BMI (kg/m^2^)		24.97 ± 2.66	24.92 ± 2.94	0.071	0.944
Dyslipidemia				-	0.287
	Yes	4 (10.3%)	0 (0%)		
	No	35 (89.7%)	21 (100.0%)		
Hypertension				0.012	0.914
	Yes	37 (94.9%)	19 (90.5%)		
	No	2 (5.1%)	2 (9.5%)		
Smoking history				2.883	0.090
	Yes	18 (46.2%)	5 (23.8%)		
	No	21 (53.8%)	16 (76.2%)		
Diabetes				0.293	0.558
	Yes	5 (12.8%)	1 (4.8%)		
	No	34 (87.2%)	20 (95.2%)		
Coronary artery disease				<0.000	>1.000
	Yes	3 (7.7%)	1 (4.8%)		
	No	36 (92.3%)	20 (95.2%)		

BMI, body mass index.

### 3.2 Comparison of Aortic Diameters Between the Two Groups

For maximum aortic root diameter, minimum aortic root diameter, maximum ascending aortic diameter, minimum ascending aortic diameter, maximum proximal aortic arch diameter, minimum proximal aortic arch diameter, maximum distal aortic arch diameter, and minimum distal aortic arch diameter, the *p*-values of the difference analysis were all less than 0.05, indicating statistically significant differences between the two groups in these variables, with the RTAD group showing smaller diameters compared to the TBAD group. For other parameters, the *p*-values of the difference analysis were all greater than 0.05, suggesting no statistically significant differences between the two groups in those variables. Details are provided in Table 2.

**Table 2.** **Comparison of aortic diameters between the two groups**.

Clinical data	TBAD (n = 39)	RTAD (n = 21)	t value	*p* value
Maximum diameter of the aortic root	37.78 ± 4.60	32.53 ± 6.47	3.639	0.001*
Minimum diameter of the aortic root	33.95 ± 4.08	23.92 ± 8.97	5.964	0.001*
Maximum diameter of the ascending aorta	35.90 ± 3.62	30.87 ± 5.83	4.125	0.001*
Minimum diameter of the ascending aorta	33.45 ± 3.29	21.41 ± 7.30	8.812	0.001*
Maximum diameter of the proximal aortic arch	35.28 ± 3.20	31.73 ± 5.26	3.247	0.002*
Minimum diameter of the proximal aortic arch	32.11 ± 2.83	22.42 ± 6.00	8.522	0.001*
Maximum diameter of the distal aortic arch	28.25 ± 3.93	24.98 ± 3.49	3.198	0.002*
Minimum diameter of the distal aortic arch	23.42 ± 3.55	18.29 ± 3.21	5.511	0.001*
Maximum diameter of the aorta proximal to the primary tear	27.67 ± 5.67	26.00 ± 4.65	1.157	0.252
Minimum diameter of the aorta proximal to the primary tear	17.86 ± 4.66	15.85 ± 4.05	1.662	0.102
Maximum diameter of the aorta at the primary tear	26.78 ± 4.82	25.78 ± 4.58	0.780	0.439
Minimum diameter of the aorta at the primary tear	17.28 ± 4.02	16.01 ± 3.97	1.170	0.247
Maximum diameter of the aorta distal to the primary tear	26.78 ± 4.04	25.15 ± 5.10	1.353	0.181
Minimum diameter of the aorta distal to the primary tear	16.46 ± 4.63	16.44 ± 3.70	0.014	0.989
Maximum diameter at the celiac trunk level	20.74 ± 2.60	20.07 ± 2.55	0.957	0.342
Minimum diameter at the celiac trunk level	13.23 ± 2.91	11.74 ± 3.70	1.717	0.091
Maximum diameter at the renal artery level	17.35 ± 1.98	17.29 ± 2.01	0.126	0.900
Minimum diameter at the renal artery level	11.99 ± 3.29	11.53 ± 3.77	0.486	0.629
Maximum diameter at the aortic bifurcation	15.85 ± 4.01	15.34 ± 2.76	0.517	0.607
Minimum diameter at the aortic bifurcation	11.62 ± 4.96	11.31 ± 4.20	0.249	0.804

Note: * indicates *p* < 0.05, indicating a statistically significant difference.

### 3.3 Comparison of Aortic True Lumen Area, False Lumen Area, Entry Tear Area, and Distance From the Tear Site to the Left Subclavian Artery Between the Two Groups

For the true lumen area of the aortic root, false lumen area of the aortic root, true lumen area of the ascending aorta, false lumen area of the ascending aorta, true lumen area of the proximal aortic arch, false lumen area of the proximal aortic arch, true lumen area of the distal aortic arch, and false lumen area of the distal aortic arch, the *p*-values of the difference analysis were all less than 0.05, indicating statistically significant differences between the two groups in these variables, with the RTAD group demonstrating higher values compared to the TBAD group. For other parameters (including entry tear area and distance from the tear site to the left subclavian artery), the *p*-values of the difference analysis were all greater than 0.05, suggesting no statistically significant differences between the two groups in those variables. Details are presented in Table 3.

**Table 3.** **Comparison of true lumen area, false lumen area, tear size, and distance from the tear to the left subclavian artery between the two groups**.

Clinical data	TBAD (n = 39)	RTAD (n = 21)	Z value	*p* value
True lumen area at the aortic root	1079.80 (850.70, 1220.80)	579.30 (419.15, 975.05)	–3.805	0.001*
False lumen area at the aortic root	0.00 (0.00, 0.00)	679.30 (505.00, 872.40)	–6.958	0.001*
True lumen area of the ascending aorta	946.40 (831.60, 1117.10)	488.30 (379.80, 793.70)	–4.673	0.001*
False lumen area of the ascending aorta	0.00 (0.00, 0.00)	702.70 (520.95, 899.65)	–7.205	0.001*
True lumen area of the proximal aortic arch	868.50 (788.90, 992.80)	541.00 (401.65, 729.15)	–4.998	0.001*
False lumen area of the proximal aortic arch	0.00 (0.00, 0.00)	463.80 (358.25, 623.65)	–7.205	0.001*
True lumen area of the distal aortic arch	519.80 (472.30, 659.30)	370.00 (270.65, 459.90)	–4.332	0.001*
False lumen area of the distal aortic arch	0.00 (0.00, 156.00)	398.60 (343.75, 496.30)	–5.804	0.001*
True lumen area proximal to the primary tear	423.00 (313.40, 493.60)	345.80 (252.95, 441.15)	–1.457	0.145
False lumen area proximal to the primary tear	442.10 (246.40, 555.30)	483.80 (406.05, 575.70)	–1.620	0.105
True lumen area at the primary tear	390.80 (300.10, 483.30)	317.50 (245.95, 399.80)	–1.806	0.071
False lumen area at the primary tear	485.10 (332.30, 636.50)	521.00 (484.30, 716.45)	–1.496	0.135
True lumen area distal to the primary tear	358.50 (278.50, 436.80)	320.40 (291.60, 393.30)	–0.628	0.530
False lumen area distal to the primary tear	564.30 (450.00, 721.20)	554.70 (440.10, 750.60)	–0.054	0.957
True lumen area at the celiac trunk level	203.60 (153.40, 265.50)	197.00 (144.95, 213.50)	–1.224	0.221
False lumen area at the celiac trunk level	229.40 (181.60, 305.40)	273.10 (228.05, 320.60)	–1.186	0.236
True lumen area at the renal artery level	156.90 (119.60, 193.60)	162.90 (101.45, 217.70)	–0.473	0.636
False lumen area at the renal artery level	151.60 (100.30, 203.80)	180.50 (145.20, 209.75)	–1.410	0.159
True lumen area at the aortic bifurcation	132.80 (100.80, 181.40)	124.70 (102.00, 164.40)	–0.457	0.648
False lumen area at the aortic bifurcation	93.20 (0.00, 167.80)	116.40 (46.65, 181.10)	–0.478	0.633
Tear size	106.00 (42.00, 159.00)	88.20 (47.25, 166.60)	–0.054	0.957
Distance from the tear to the left subclavian artery	–24.00 (–29.00, –16.00)	–21.00 (–33.00, –9.50)	–0.706	0.480

Note: * indicates *p* < 0.05, indicating a statistically significant difference.

### 3.4 Comparison of Aortic Arch Type and Tear Location Between the Two Groups

The *p*-values of the difference analysis for both aortic arch type and tear location were greater than 0.05, indicating no statistically significant differences between the two groups in these variables. Details are provided in Table 4.

**Table 4.** **Comparison of aortic arch types and tear locations**.

Clinical data		TBAD (n = 39)	RTAD (n = 21)	χ^2^ value	*p* value
Aortic arch morphology				0.152	0.927
	Type I Arch	14 (35.9%)	8 (38.1%)		
	Type II Arch	20 (51.3%)	11 (52.4%)		
	Type III Arch	5 (12.8%)	2 (9.5%)		
Tear location				4.742	0.192
	Anterior Wall	3 (7.7%)	5 (23.8%)		
	Posterior Wall	1 (2.6%)	2 (9.5%)		
	Lesser Curvature	11 (28.2%)	5 (23.8%)		
	Greater Curvature	24 (61.5%)	9 (42.9%)		

### 3.5 Comparison of Aortic Arch Angles Between the Two Groups

The *p*-values of the difference analysis for both the alpha and beta angles of the aortic arch were greater than 0.05, indicating no statistically significant differences between the two groups in these variables. Details are presented in Table 5.

**Table 5.** **Comparison of aortic arch angles**.

Clinical data	TBAD (n = 39)	RTAD (n = 21)	t value	*p* value
α Angle	152.10 ± 11.05	153.86 ± 11.94	–0.570	0.571
β Angle	128.15 ± 10.68	131.81 ± 7.53	–1.390	0.170

### 3.6 LASSO Regression Analysis

**Fig. 4.**
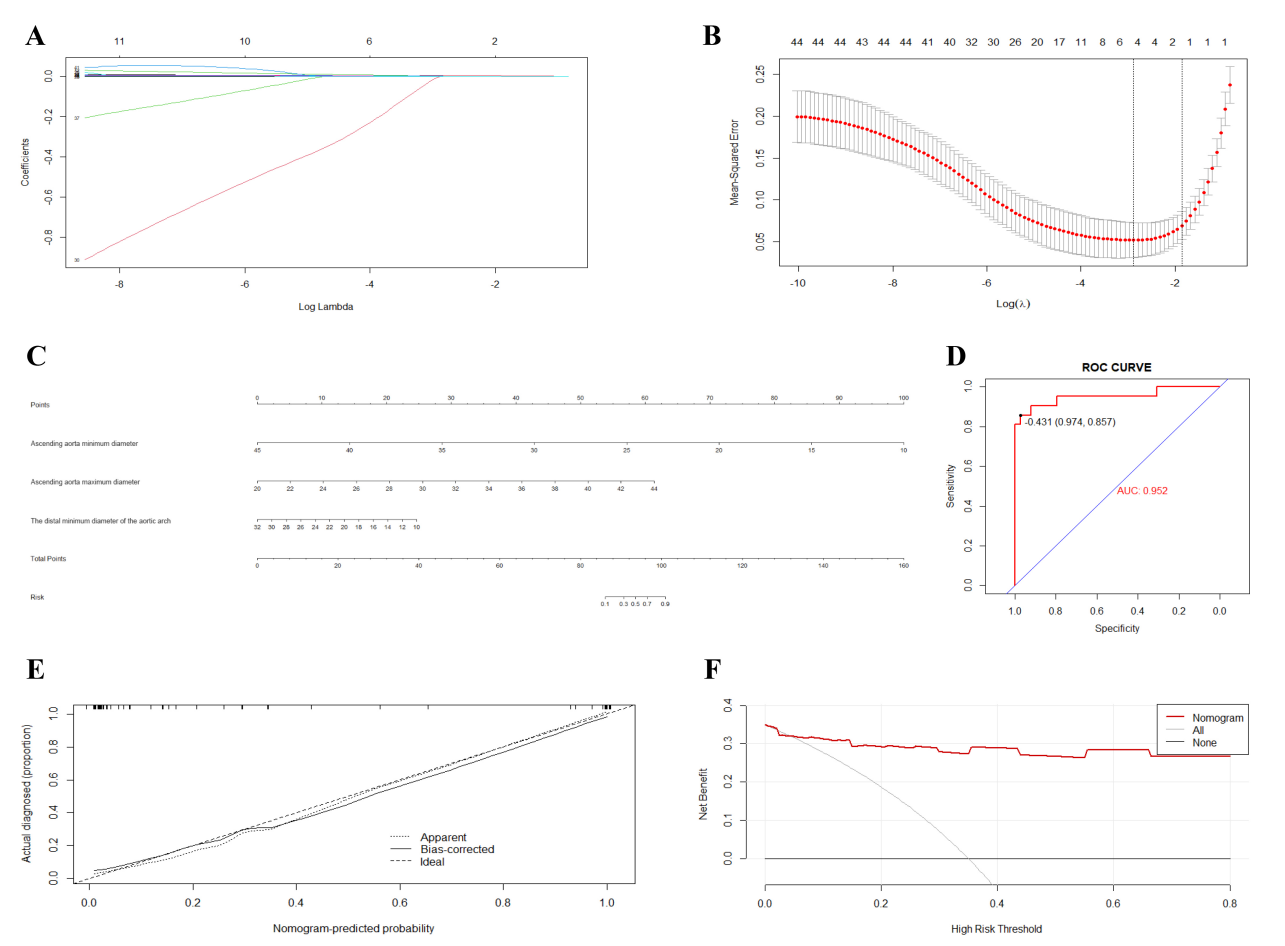
**LASSO regression results for 44 predictor variables**. (A) LASSO Dimension Reduction Results. (B) Process of selecting optimal independent variables via ten-fold cross-validation and identifying the optimal λ value through LASSO cross-validation. (C) Risk Prediction Model for RTAD Occurrence. (D) ROC/AUC Evaluation of the Prediction Model. (E) Validation of the Prediction Model Robustness. (F) Evaluation of the Prediction Model. Note: Fig. 4A displays the coefficient trajectories of the 44 candidate independent variables. Fig. 4B illustrates the optimal variable selection via LASSO regression with 10-fold cross-validation. Following dimensionality reduction, the retained variables for subsequent analysis include: Minimum ascending aortic diameter (partial regression coefficient: 0.0001503664); Maximum ascending aortic diameter (partial regression coefficient: 0.0008316777); Maximum distal aortic arch diameter (partial regression coefficient: 0.00005818305); Minimum distal aortic arch diameter (partial regression coefficient: 0.0001007072); Proximal aortic arch true lumen area (partial regression coefficient: –0.00000000100702); Constant term: 0.08353068. LASSO, Least Absolute Shrinkage and Selection Operator; ROC, receiver operating characteristic; AUC, area under the curve.

A total of 44 independent variables were included in this study. Dimensionality reduction was performed to screen for the most representative high-risk predictors through LASSO regression analysis. The λ value was selected as the optimal one through ten-fold cross-validation using the 1-SE criterion, rather than being manually preset. As the penalty coefficient (λ) increased, the coefficients of initially included variables were gradually shrunk, with some ultimately compressed to zero, thereby preventing model overfitting. The optimal penalty coefficient (λ) was identified using 10-fold cross-validation based on the minimum criterion. When λ increased to the value corresponding to one standard error (λ = 1 SE), it was selected as the optimal value for the model. Results are detailed below (Fig. 4A,B).

### 3.7 Multivariable Logistic Regression

The results of the multivariable logistic regression analysis for the selected predictors are as follows:

(1) Minimum ascending aortic diameter: The regression test *p*-value was less than 0.05, indicating a statistically significant influence on the dependent variable when controlling for other covariates. The odds ratio (OR) was less than 1 (OR <1), suggesting that an increase in this parameter was associated with a reduced incidence of RTAD outcomes, thus identified as an independent protective factor. (2) Maximum ascending aortic diameter: The regression test *p*-value was less than 0.05, demonstrating a statistically significant effect. The OR was greater than 1 (OR >1), indicating that an increase in this parameter correlated with an elevated incidence of RTAD outcomes, categorizing it as an independent risk factor. (3) Maximum distal aortic arch diameter: The regression test *p*-value exceeded 0.05, showing no statistically significant impact on the dependent variable after adjusting for confounders. (4) Minimum distal aortic arch diameter: The regression test *p*-value was less than 0.05, confirming statistical significance. The OR was less than 1 (OR <1), signifying that an increase in this measurement reduced the likelihood of RTAD outcomes, establishing it as an independent protective factor. (5) Proximal aortic arch true lumen area: The regression test *p*-value was greater than 0.05, indicating no statistically significant association with the outcome variable. Detailed results are summarized in Table 6.

**Table 6.** **Multivariate logistic regression analysis of independent variables after LASSO dimension reduction**.

Variable	β	Standard	Wald	*p*	OR	95% CI for OR	
		Error				Lower	Upper
Minimum diameter of the ascending aorta	–0.717	0.352	4.141	0.042*	0.488	0.245	0.974
Maximum diameter of the ascending aorta	0.841	0.377	4.965	0.026*	2.318	1.107	4.857
Maximum diameter of the distal aortic arch	0.272	0.184	2.188	0.139	1.313	0.915	1.884
Minimum diameter of the distal aortic arch	–0.521	0.252	4.257	0.039*	0.594	0.362	0.974
True lumen area of the proximal aortic arch	–0.007	0.007	0.963	0.326	0.993	0.979	1.007
Constant	1.207	6.177	0.038	0.845	3.345		

Note: Hosmer-Lemeshow = 13.310, *p* = 0.102 > 0.05, indicating the model is valid (*p < 0.05 indicates statistical significance).

### 3.8 Development of the Clinical Prediction Model in the Test Set

Fig. 4C presents the nomogram constructed using statistically significant variables from the logistic regression equation to predict the risk of RTAD occurrence. In the nomogram: (1) The Points scale (first row) provides a scoring reference for each variable. (2) For any individual patient, the corresponding Points can be assigned based on the value or category of each predictor variable. (3) Total Points are calculated by summing the individual points for all variables. (4) The Total Points are then mapped to the Risk scale (second-to-last row) to obtain the predicted probability of RTAD occurrence.

### 3.9 Evaluation of the Clinical Prediction Model

#### 3.9.1 C-index

The concordance index (C-index) was 0.952 (SD = 0.0068), with a Z-statistic of 13.400 and a *p*-value of 0.001 (<0.05). These results confirm statistically significant concordance, indicating excellent predictive performance of the model.

#### 3.9.2 ROC Curve

As shown in the ROC curve below, the area under the curve (AUC) was 0.952 (95% confidence interval: 0.819–1.000), with a sensitivity of 85.7% and specificity of 97.4%, further demonstrating the model’s strong predictive capability. Details are provided in Fig. 4D.

#### 3.9.3 Calibration Curve

Internal validation was performed using the Bootstrap resampling method with 1000 iterations to assess the model’s robustness. The calibration curve (Fig. 4E) demonstrates slight fluctuations between the observed model fit, expected model fit, and bias-corrected estimates, which may be attributable to the limited sample size. Overall, the model demonstrated good robustness and is generalizable for clinical application.

#### 3.9.4 Decision Curve Analysis (DCA)

In combination with the ROC curve, decision curve analysis (DCA) was employed to evaluate the clinical utility of the prediction model. The DCA results further confirmed the robustness and practical applicability of our model. Compared with the “treat-all” and “treat-none” strategies, the model demonstrated a higher net benefit across the entire range of threshold probabilities, indicating good stability and reliability under different clinical decision thresholds. These findings suggest that the model has the potential to serve as an important tool for individualized RTAD risk stratification and clinical decision support. Detailed results are presented in Fig. 4F.

## 4. Discussion

### 4.1 High-Risk Factors for Aortic Dissection

Aortic dissection (AD) is a life-threatening cardiovascular disease. Over the years, researchers have focused on identifying its high-risk factors. Studies have reported that age, hypertension, dyslipidemia, and inherited connective tissue disorders are significant risk factors for AD [22]. Additionally, aortic geometric morphology has been shown to play a critical role in the development of both Type A and Type B aortic dissections.

### 4.2 Risk Factors for TAAD

In TAAD, specific geometric features of the proximal aorta, including elongation, angulation, and tortuosity, may significantly influence pathogenesis [23, 24, 25, 26, 27, 28]. Ascending aortic diameter remains the only widely accepted morphological risk factor for TAAD, with prophylactic surgery recommended when the diameter exceeds 55 mm, even in asymptomatic patients [29]. However, Krüger *et al*. [23] argue that aortic diameter alone may not be the optimal predictor, as most dissections occur at diameters below 55 mm. They propose aortic elongation as a potential risk factor for TAAD [23].

### 4.3 Risk Factors for TBAD

Studies on TBAD highlight increased aortic arch curvature and angulation as independent and specific predictors [30]. Other factors, such as aortic elongation, incremental angulation, and tortuosity indices, are also associated with TBAD [31, 32]. However, spatial geometric measurements on CTA are complex and time-consuming, complicating risk assessment. Sun *et al*. [33] proposed an easily identifiable morphological parameter—aortic arch type—as a surrogate marker. Type III aortic arch, characterized by elongation, increased angulation, and tortuosity, serves as a comparable identifier for high-risk TBAD patients [33].

### 4.4 Risk Factors for RTAD

Previous studies have broadly focused on geometric features of AD or compared TAAD and TBAD. However, RTAD, a distinct subtype, remains understudied. DiMusto *et al*. [20] demonstrated that RTAD often occurs when the primary entry tear is near the aortic arch with poor false lumen decompression through distal branches. This may result from increased pressure in the false lumen due to thrombus formation or slow flow, leading to retrograde propagation into the ascending aorta. One possible reason is that the patient population we studied and the models or data used in the studies by DiMusto *et al*. [20] differ. For example, our study only included primary RTAD, while the studies by DiMusto *et al*. [20] may have covered different types of aortic dissection, and the sample size and population characteristics may also have been different. In addition, our study emphasized the relationship between changes in aortic geometry and the occurrence of RTAD, while the studies by DiMusto *et al*. [20] mainly focused on the impact of the tear location. Therefore, different research focuses may have led to these differences. Osswald *et al*. [21] identified elevated wall shear stress (WSS) at the subclavian artery distal region in RTAD patients using computational fluid dynamics, suggesting WSS as a potential screening marker when combined with clinical risk factors. Dziodzio *et al*. [19] highlighted the importance of primary entry tear location in porcine models, noting that tears on the aortic arch’s lesser curvature may predispose to RTAD. This study focuses on primary RTAD, distinct from iatrogenic or stent-graft-induced RTAD, emphasizing differences in pathophysiology compared to TBAD and TAAD.

### 4.5 RTAD Risk Prediction Model

This study identifies reduced minimum ascending aortic diameter, increased maximum ascending aortic diameter, and reduced minimum distal aortic arch diameter as specific predictors for RTAD development in TBAD patients. These geometric changes may increase vascular eccentricity, altering hemodynamics and wall shear stress to influence dissection propagation. Although Dziodzio *et al*. [19] linked RTAD risk to primary tear location on the lesser curvature, this study found no such association, possibly due to anatomical differences between porcine and human aortas. Additionally, post-TEVAR RTAD risk may correlate with aortic arch angulation and curvature [34], but no significant differences in α/β angles were observed here, likely due to distinctions between primary and post-TEVAR RTAD.

The RTAD risk prediction nomogram, validated by a C-index of 0.952, ROC-AUC of 0.952, and robust calibration/DCA curves, provides clinical guidance for early intervention. High-risk patients should undergo intensified screening to mitigate retrograde progression risks.

### 4.6 Study Limitations

This single-center retrospective study is limited by potential measurement variability despite standardized protocols. The small sample size and selection bias inherent to retrospective designs challenge generalizability. Future multi-center prospective studies with long-term follow-up are needed to validate these findings.

## 5. Conclusions

(1) Morphological Differences: Despite sharing entry tear locations, RTAD and TBAD exhibit distinct morphological progression mechanisms. Reduced minimum ascending aortic diameter, increased maximum ascending aortic diameter, and reduced minimum distal aortic arch diameter may serve as specific predictors for RTAD development in TBAD patients. (2) Clinical Utility: The RTAD risk prediction model, incorporating these unique factors, offers actionable insights for preventive strategies and early clinical intervention, potentially improving outcomes through targeted screening and management.

## Availability of Data and Materials

The datasets used in this study are available from the corresponding author upon reasonable request.
